# Predictors of failure of early neurological improvement in early time window following endovascular thrombectomy: a multi-center study

**DOI:** 10.3389/fneur.2023.1227825

**Published:** 2023-09-14

**Authors:** Yuzheng Lai, Francesco Diana, Mohammad Mofatteh, Thanh N. Nguyen, Eric Jou, Sijie Zhou, Hao Sun, Jianfeng He, Wenshan Yan, Yiying Chen, Mingzhu Feng, Junbin Chen, Jicai Ma, Xinyuan Li, Heng Meng, Mohamad Abdalkader, Yimin Chen

**Affiliations:** ^1^Department of Neurology, Guangdong Provincial Hospital of Integrated Traditional Chinese and Western Medicine (Nanhai District Hospital of Traditional Chinese Medicine of Foshan City), Foshan, China; ^2^Department of Neuroradiology, A.O.U. San Giovanni di Dio e Ruggi d’Aragona, University of Salerno, Salerno, Italy; ^3^School of Medicine, Dentistry and Biomedical Sciences, Queen’s University Belfast, Belfast, United Kingdom; ^4^Department of Radiology, Boston University Chobanian and Avedisian School of Medicine, Boston, MA, United States; ^5^School of Clinical Medicine, University of Cambridge, Cambridge, United Kingdom; ^6^Department of Surgery of Cerebrovascular Diseases, First People’s Hospital of Foshan, Foshan, China; ^7^Department of Neurology and Advanced National Stroke Center, Foshan Sanshui District People’s Hospital, Foshan, China; ^8^Department of Neurology, The Affiliated Yuebei People’s Hospital of Shantou University Medical College, Shaoguan, China; ^9^The Second School of Clinical Medicine, Southern Medical University, Guangzhou, China; ^10^Department of Neurology, The First Affiliated Hospital of Jinan University, Clinical Neuroscience Institute of Jinan University, Guangzhou, China; ^11^Neuro International Collaboration (NIC), Foshan, China

**Keywords:** early neurological improvement, early time window, endovascular therapy, ischemic stroke, patient outcome

## Abstract

**Background and objective:**

Endovascular thrombectomy (EVT) has become the gold standard in the treatment of acute stroke patients. However, not all patients respond well to this treatment despite successful attempts. In this study, we aimed to identify variables associated with the failure of improvements following EVT.

**Methods:**

We retrospectively analyzed prospectively collected data of 292 ischemic stroke patients with large vessel occlusion who underwent EVT at three academic stroke centers in China from January 2019 to February 2022. All patients were above 18 years old and had symptoms onset ≤6 h. A decrease of more than 4 points on the National Institute of Health Stroke Scale (NIHSS) after 24 h compared with admission or an NIHSS of 0 or 1 after 24 h was defined as early neurological improvement (ENI), whereas a lack of such improvement in the NIHSS was defined as a failure of early neurological improvement (FENI). A favorable outcome was defined as a modified Rankin scale (mRS) score of 0–2 after 90 days.

**Results:**

A total of 183 patients were included in the final analyses, 126 of whom had FENI, while 57 had ENI. Favorable outcomes occurred in 80.7% of patients in the ENI group, in contrast to only 22.2% in the FENI group (*p* < 0.001). Mortality was 7.0% in the ENI group in comparison to 42.1% in the FENI group (*p* < 0.001). The multiple logistic regression model showed that diabetes mellitus [OR (95% CI), 2.985 (1.070–8.324), *p* = 0.037], pre-stroke mRS [OR (95% CI), 6.221 (1.421–27.248), *p* = 0.015], last known well to puncture time [OR (95% CI), 1.010 (1.003–1.016), *p* = 0.002], modified thrombolysis in cerebral infarction = 3 [OR (95% CI), 0.291 (0.122–0.692), *p* = 0.005], and number of mechanical thrombectomy passes [OR (95% CI), 1.582 (1.087–2.302), *p* = 0.017] were the predictors of FENI.

**Conclusion:**

Diabetes mellitus history, pre-stroke mRS, longer last known well-to-puncture time, lack of modified thrombolysis in cerebral infarction = 3, and the number of mechanical thrombectomy passes are the predictors of FENI. Future large-scale studies are required to validate these findings.

## 1. Introduction

Stroke is one of the major causes of global mortality and patient disability (1). Treatment of stroke has improved significantly over the past few decades, and endovascular thrombectomy (EVT) has become the gold standard for the treatment of acute ischemic stroke (AIS) (2, 3). However, a significant proportion of AIS patients do not benefit from EVT despite successful attempts (4).

Multiple studies have reported that early neurological improvements (ENI) can be used as a surrogate in predicting functional outcomes of AIS patients (5–10). For example, a decrease in the National Institute of Health Stroke Scale (NIHSS) score of greater than 4 points or NIHSS was 0 or 1 at 24 h after EVT, defined as ENI, was considered as a surrogate of long-term outcomes after mechanical thrombectomy (11). Conversely, a failure of early neurological improvement (FENI) was defined as an unchanged or decrease in the NIHSS score <4 points at 24 h after EVT (11). However, variables that can predict early ENI within 24 h after EVT remain understudied (11, 12). Identifying markers of early neurological improvement can be important in predicting longer-term outcomes in patients with large vessel occlusion undergoing endovascular therapy. The objective of this study was to investigate factors that contribute to ENI in patients with large vessel occlusion presenting in the early time window following EVT.

## 2. Materials and methods

### 2.1. Patients

We included consecutive patients with AIS who underwent EVT from January 2019 to February 2022 at three academic comprehensive stroke centers in China. We used data from the Big Data Observatory Platform for stroke in China, a prospectively collected data platform registry for stroke patients following EVT and the hospital data platforms. The inclusion criteria were as follows: (1) patients with AIS who underwent EVT; (2) age ≥18 years old at the time of treatment; (3) symptom onset ≤6 h. The exclusion criteria were as follows: (1) pre-EVT NIHSS <6; (2) missing data of follow-up; (3) symptoms onset >6 h.

### 2.2. Study outcome and assessment

Patients were divided into two groups according to their evolution of neurological deficits. A decrease of more than 4 points on the National Institute of Health Stroke Scale (NIHSS) after 24 h compared with admission or an NIHSS of 0 or 1 after 24 h was defined as early neurological improvement (ENI), whereas a lack of such improvement in the NIHSS was defined as a failure of early neurological improvement (FENI). We collected the following data: age, sex, vascular risk factors, premorbid modified Rankin scale (mRS), door-to-needle time (DNT), onset-to-needle time (ONT), door-to-puncture time (DPT), last known well-to-puncture time (LKWPT), door to recanalization time (DRT), puncture to recanalization (PRT), site of arterial occlusion, number of thrombectomy passes, and modified thrombolysis in cerebral infarction (mTICI) post-thrombectomy. Successful reperfusion was defined as an mTICI of ≥2B, and complete reperfusion was defined as an mTICI of 3. Experienced attending neurologists measured and recorded the NIHSS of all consecutive patients at the admission, while the interventionist recorded the NIHSS after the EVT. Patients’ outcomes were evaluated by mRS at 90 days. Stroke nurses and/or the attending neurology physicians routinely assessed the mRS during in-person appointments or remotely by telephone during outpatient follow-up. A favorable outcome was defined as an mRS of 0–2 after 3 months, and an mRS of 6 was defined as mortality.

### 2.3. Statistical analysis

IBM SPSS version 26 was performed to analyze and draft the figure. The Mann–Whitney *U*-test was performed for non-normally distributed continuous data, recorded as medians along with the interquartile range (IQR). Normally distributed variables were reported as means with corresponding standard deviations (SD) and compared using the student’s *t*-test. Results were considered statistically significant if the *p*-value was <0.05.

### 2.4. Ethics

The study protocol was approved by the Guangdong Provincial Hospital of Integrated Traditional Chinese and Western Medicine, Guangdong Province, China. Informed consent was not required due to the retrospective nature of the study in compliance with national laws and regulations. All procedures performed in the studies involving human participants were in accordance with the ethical standards of the institutional and/or national research committee and with the 1964 Declaration of Helsinki and its later amendments or comparable ethical standards.

## 3. Results

From January 2019 to February 2022, we treated 292 consecutive AIS patients who had large vessel occlusion and underwent EVT. A total of 103 patients were treated with an LKWPT >360 min, four patients were lost to follow-up, one had a pre-EVT NIHSS <6, and one did not have the 24 h NIHSS documented. Therefore, 183 patients were included in the final analysis. Among them, 126 patients had FENI, while 57 had ENI.

The baseline characteristics of patients in both groups are demonstrated in Table 1. There were no statistically significant differences between baseline characteristics of the two groups including age (*p* = 0.031) and sex (*p* = 0.214), as well as risk factors, such as hypertension (*p* = 0.321), diabetes mellitus (*p* = 0.106), coronary artery disease (*p* = 0.545), atrial fibrillation (*p* = 0.641), stroke history (*p* = 0.791), and chronic kidney disease (*p* = 0.696). Smoking was less frequent in the FENI group compared to the ENI group (12.0% vs. 24.6%, *p* = 0.030). In addition, NIHSS at admission [FENI 15 (12–19) vs. ENI 14 (10–18), *p* = 0.15], premorbid mRS (FENI 0 vs. ENI 0, *p* = 0.051), baseline Alberta stroke program early CT score (ASPECTS) [FENI 8 (8–9) vs. ENI 8 (8–9), *p* = 0.362], and distribution of target vessel occlusion side (*p* = 0.11) were similar in both groups.

**Table 1:** Comparison of baseline and failure of early neurological improvement (FENI) and early neurological improvement (ENI) groups.

Baseline characteristics	FENI (*n* = 126)	ENI (*n* = 57)	*X*^2^/*t*/*z*	*p*
Age ± SD	66.7 ± 12.12	62.22 ± 13.47	2.179	0.031
Female, *n*, %	39 (30.95)	23 (40.35)	1.548	0.214
*Risk factors*				
Hypertension, *n*, %	74 (58.73)	29 (50.88)	0.984	0.321
Diabetes mellitus, *n*, %	31 (24.60)	8 (14.04)	2.614	0.106
Coronary artery disease, *n*, %	27 (21.43)	10 (17.54)	0.367	0.545
Atrial fibrillation, *n*, %	51 (40.48)	21 (36.84)	0.217	0.641
Prior stroke, *n*, %	18 (14.29)	9 (15.79)	0.071	0.791
Hyperlipemia, *n*, %	22 (17.46)	10 (17.54)	0.001	0.989
Chronic kidney disease, *n*, %	11 (8.73)	4 (7.02)	0.153	0.696
Smoker, *n*, %	15 (11.90)	14 (24.56)	4.714	0.030^*^
NIHSS pre-EVT (IQR)	15.00 (12.00, 19.00)	14.00 (10.00, 18.00)	−1.445	0.148
ASPECTS pre-treatment (IQR)	8.00 (8.00, 9.00)	8.00 (8.00, 9.00)	−0.912	0.362
mRS pre-premorbid (IQR)	0.00 (0.00, 0.00)	0.00 (0.00, 0.00)	−1.949	0.051
*Occlusion vascular cites*				
Distal/terminal ICA, *n*, %	25 (19.84)	6 (10.53)	8.982	0.110
MCA-M1, *n*, %	45 (35.71)	31 (54.39)		
MCA-M2, *n*, %	12 (9.52)	2 (3.51)		
Tandem, *n*, %	24 (19.05)	8 (14.04)		
Basilar, *n*, %	14 (11.11)	5 (8.77)		
Others, *n*, %	6 (4.76)	5 (8.77)		
*Technical outcomes*				
IV thrombolysis, *n*, %	67 (53.17)	30 (52.63)	0.005	0.946
DNT	48.00 (31.00, 60.00)	41.00 (29.50, 51.00)	−1.073	0.283
ONT	136.00 (100.50, 177.00)	115.00 (90.50, 174.50)	−0.855	0.393
DRT (IQR), min	230.00 (169.75, 295.00)	195.00 (149.50, 240.00)	−2.819	0.005^*^
DPT (IQR), min	143.50 (105.00, 190.00)	123.00 (98.00, 159.50)	−1.633	0.102^*^
PRT (IQR), min	75.50 (47.50, 114.25)	49.00 (33.50, 78.50)	−3.264	0.001^*^
LKNPT (IQR), min	251.50 (183.75, 299.25)	200.00 (136.50, 265.50)	−2.791	0.005^*^
mTICI post ≥2B, *n*, %	96 (76.19)	54 (94.74)	9.133	0.003^*^
mTICI post = 3	42 (33.33)	35 (61.40)	12.688	<0.001^*^
sICH, *n*, %	22 (17.46)	0 (0.00)	11.312	0.001^*^
No. of EVT passes	2.00 (1.00, 3.00)	1.00 (1.00, 2.00)	−2.411	0.016^*^

^*^The significance level was defined as a *p*-value of <0.05. ASPECTS, the Alberta stroke program early CT score; DPT, door-to-puncture time; DNT, door-to-needle time; DRT, door-to-recanalization time; ENI, early neurological improvement; EVT, endovascular thrombectomy; FENI, failure of early neurological improvement; LKNPT, last-known normal-to-puncture time; ICA, internal carotid artery; IQR, interquartile range; IV, intravenous; MCA, middle cerebra artery; mRS, modified Rankin scale; mTICI, modified thrombolysis in cerebral infarction; NIHSS, National Institute of Health Stroke Scale; ONT, onset-to-needle time; SD, standard deviation; SICH, symptomatic intracranial hemorrhage.

The rate of bridging intravenous (IV) thrombolysis before EVT was similar (FENI 53.2% vs. ENI 52.6%, *p* = 0.946) between the two groups. Patients with FENI required more attempts during EVT [2 (1–3) vs. 1 (1–2); *p* = 0.016], had longer last-known well-to-puncture time [251.50 min (183.75–299.25) vs. 200 min (136.50–265.50); *p* = 0.005] and door to recanalization time [230 min (169.75–295) vs. 195 min (149.50–240); *p* = 0.005]. Successful reperfusion defined by modified thrombolysis in cerebral infarction ≥2B was lower in the FENI compared to the ENI group [76.2% vs. 94.7%; *p* = 0.003]. Furthermore, symptomatic intracranial hemorrhage (sICH) occurred in 14.5% of patients with FENI, and there were no patients with ENI (*p* = 0.001) after EVT (Table 1).

**Figure 1:**
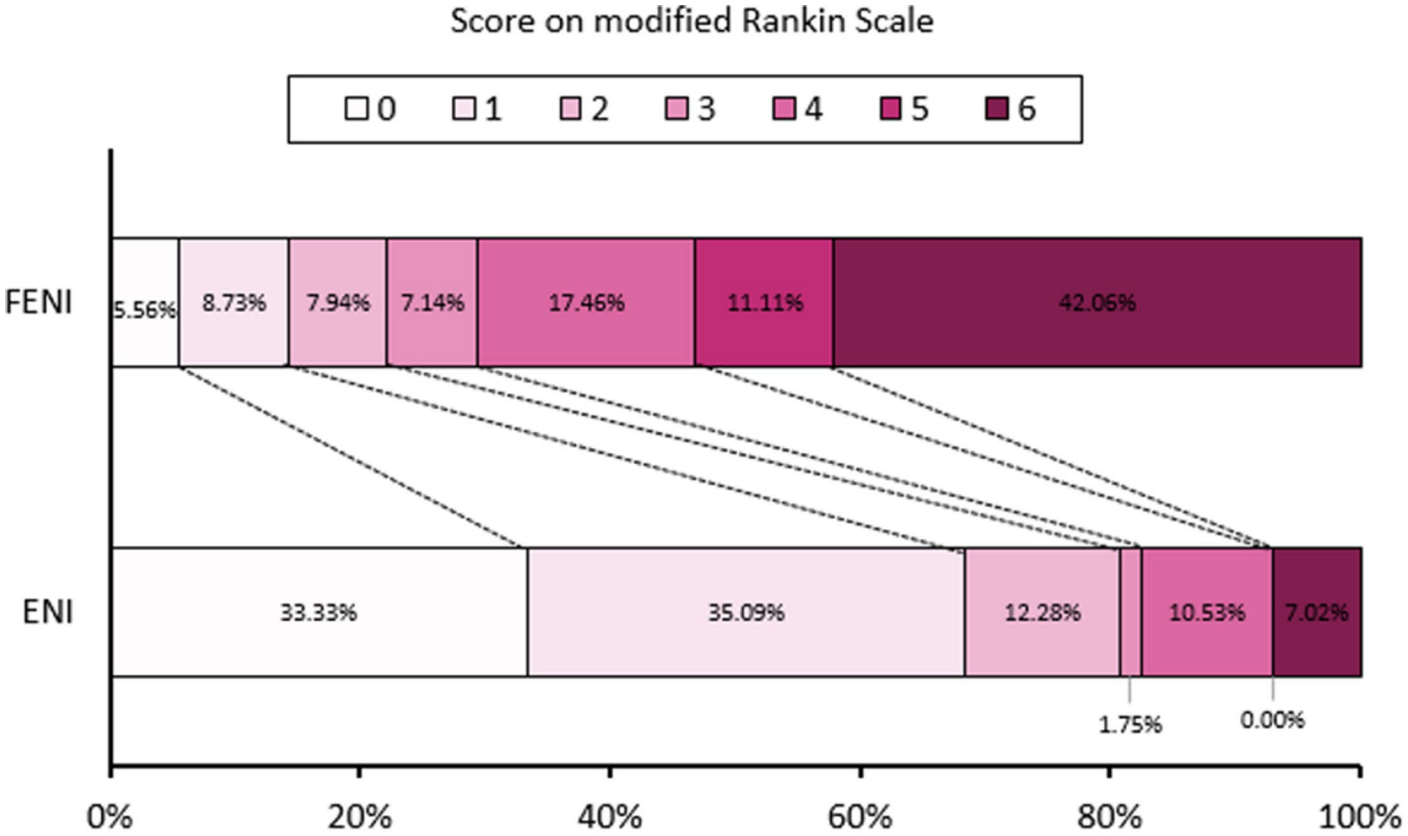
Distribution of 90 days modified Rankin scale (mRS) of failure of early neurological improvement (FENI) and early neurological improvement (ENI).

Functional outcome was significantly different between the two groups (Table 2). Patients with ENI were more likely to experience a good outcome at discharge [median mRS 5 (3–5) in the FENI group vs. 2 (1–3) in the ENI group, *p* < 0.001] and at the 3 months follow-up (mRS of 0–2 in 22.2% of FENI vs. 80.7% of ENI, and mRS of 6 in 42.1% of FENI vs. 7% of ENI; *p* < 0.001). Furthermore, mortality was 7.0% (*n* = 4) in the ENI group in comparison to 42.1% (*n* = 53) in the FENI group (*p* < 0.001). The distribution of 90 days mRS of FENI and ENI groups is shown in Figure 1 and Table 3.

**Table 2:** Comparison of mRS at discharge, 90 days favorable outcome and mortality in failure of early neurological improvement (FENI), and early neurological improvement (ENI) groups.

Outcome	FENI, *N* = 126 (%)	ENI, *N* = 57 (%)	*X*^2^/*z*	*p*
mRS discharge (IQR)	5.00 (3.00, 5.00)	2.00 (1.00, 3.00)	−7.646	<0.001
90 days favorable outcome, *n*, %	28 (22.22)	46 (80.70)	55.724	<0.001
90 days mortality, *n*, %	53 (42.06)	4 (7.02)	22.476	<0.001

ENI, early neurological improvement; FENI, failure of early neurological improvement; IQR, interquartile range; mRS, modified Rankin scale.

**Table 3:** Distribution of 90 days mRS of FENI and ENI.

	FENI	ENI	*X*^2^/*t*/*z*	*p*
90 days mRS of 0	7 (5.56)	19 (33.33)	63.334	<0.001
90 days mRS of 1	11 (8.73)	20 (35.09)		
90 days mRS of 2	10 (7.94)	7 (12.28)		
90 days mRS of 3	9 (7.14)	1 (1.75)		
90 days mRS of 4	22 (17.46)	6 (10.53)		
90 days mRS of 5	14 (11.11)	0 (0.00)		
90 days mRS of 6	53 (42.06)	4 (7.02)		

ENI, early neurological improvement; FENI, failure of early neurological improvement; mRS, modified Rankin scale.

Differences in outcomes between ENI and FENI groups, including 90 days favorable outcome (*p* < 0.001) and 90 days mortality (*p* < 0.001) were present after adjusting for confounding factors, including age, current smoking status, diabetes mellitus, NIHSS pre-EVT, and premorbid mRS (Table 4).

**Table 4:** Outcome of ENI vs. FENI after adjusting the confounding factor.

	Univariable analysis OR (95% CI), *p*-value	Multivariate analysis OR (95% CI), *p*-value
90 days favorable outcome, *n*, %	14.636 (6.706–31.944), <0.001	15.555 (6.384–37.901), <0.001
90 days mortality, *n*, %	0.104 (0.035–0.305), <0.001	0.110 (0.035–0.346), <0.001

Adjusted factors were age, current smoker, diabetes, pre-endovascular thrombectomy, National Institute of Health Stroke Scale, and premorbid modified Rankin scale (mRS).

Next, we decided to identify variables that can predict FENI outcomes. Eleven variables were assessed in a multiple logistic regression model, showing that diabetes mellitus [OR (95% CI), 2.985 (1.070–8.324), *p* = 0.037], pre-stroke mRS [OR (95% CI), 6.221 (1.421–27.248), *p* = 0.015], longer last known well to puncture time [OR (95% CI), 1.010 (1.003–1.016), *p* = 0.002], lack of modified thrombolysis in cerebral infarction = 3 [OR (95% CI), 0.291 (0.122–0.692), *p* = 0.005], and the number of mechanical thrombectomy passes [OR (95% CI), 1.582 (1.087–2.302), *p* = 0.017] were predictors of FENI (Table 5).

**Table 5:** Independent predictors of FENI by logistic regression analysis.

Variable	OR	95% CI	*p*
Age	1.025	0.995–1.057	0.107
Current smoker	0.562	0.202–1.560	0.268
Diabetes mellitus	2.985	1.070–8.324	0.037^*^
mRS premorbid	6.221	1.421–27.248	0.015^*^
DPT	0.997	0.986–1.007	0.517
DRT	1.001	0.993–1.008	0.857
Last known well to puncture time	1.010	1.003–1.016	0.002^*^
mTICI post ≥2B	0.351	0.074–1.671	0.189
mTICI post = 3	0.291	0.122–0.692	0.005^*^
No. of EVT passes	1.582	1.087–2.302	0.017^*^

^*^The significance level was defined as a *p*-value of <0.05. DPT, door-to-puncture time; DRT, door-to-recanalization time; EVT, endovascular thrombectomy; mTICI, modified thrombolysis in cerebral infarction.

## 4. Discussion

This study analyzed the clinical variables for their association with FENI in patients with AIS due to LVO who underwent EVT. We found that diabetes history, pre-stroke mRS, LKWPT, absence of complete (TICI 3) reperfusion, number of MT passes, and ICH were independent predictors of FENI. Predictors of FENI were consistent with predictors of reperfusion without functional independence (RFI), a frequent phenomenon that may occur in the extended time window. Indeed, a multi-center cohort study recently demonstrated that higher baseline NIHSS, higher pre-stroke disability, transfer to a comprehensive stroke center, and a longer interval to puncture were independent predictors of RFI in patients with late presentation AIS (13). Conversely, in agreement with previous studies (12, 14), we showed that ENI was strongly associated with good functional outcomes, low rates of sICH, and reduced mortality. Thus, we may argue that predictors of FENI can be considered surrogate parameters of long-term clinical outcomes in patients with AIS who underwent MT. Recognizing such factors can help to identify patients who may be at risk of FENI and establish future studies to circumvent these limitations.

As previously demonstrated in multiple studies (15–18), diabetes mellitus did not influence recanalization; nevertheless, it was a predictor of poor outcomes at 90 days. Moreover, a higher admission glucose value was an independent predictor of sICH (17). As recanalization rates do not explain the worse outcome of patients with diabetes mellitus, the mechanisms accounting for this are likely to be on the capillary, cellular, or metabolic level. Potential mechanisms leading to more extensive neuronal cell damage and worse outcomes in stroke patients with higher glucose levels include accumulation of extracellular glutamate (18), intracellular acidosis, increased blood-brain barrier disruption (19, 20), and formation of brain edema (21).

Furthermore, longer last known normal to puncture time was an independent predictor of FENI and strategies to optimize treatment time are critical to improving patient outcomes (22, 23).

.The limitations of this study are related to the retrospective design. Furthermore, uncertainty might arise because of the imputation of missing values. A confirmation and analysis of independent predictors of FENI can be achieved with larger, multi-center cohort studies. Despite such limitations, our findings can help identify factors that can predict the successful outcome of EVT, which can provide pragmatic points for randomized controlled trials, facilitate surrogacy for patients who are unlikely to follow-up, and establish predictive models for ischemic stroke patients’ outcomes (24).

## 5. Conclusion

Early neurological improvement was a reliable surrogate to predict 90 days favorable outcomes and mortality. Diabetes, premorbid mRS, longer last known well time to puncture time, absence of mTICI 3, sICH, and more EVT passes predicted the failure of early neurological improvement in the early time window following endovascular therapy. Future randomized controlled trials are required to validate these findings.

## Data availability statement

The raw data supporting the conclusions of this article will be made available by the authors, without undue reservation.

## Ethics statement

The studies involving humans were approved by Foshan Sanshui District People’s Hospital in Foshan. The studies were conducted in accordance with the local legislation and institutional requirements. Written informed consent for participation was not required from the participants or the participants’ legal guardians/next of kin because informed consents were not required due to the retrospective nature of the study in compliance with national laws and regulations.

## Author contributions

FD, YnC, YL, and HM drafted the manuscript. MM, MA, and TN critically edited the manuscript. EJ, SZ, HS, JH, WY, YgC, MF, JC, JM, and XL analyzed the cases and edited the manuscript. All authors contributed to the article and approved the submitted version.

## Funding

The study was supported by the Foshan Science and Technology Bureau (Grant No. 2220001005022), the Medical Science and Technology Research Foundation of Guangdong Province (Grant No. 20221027164016611), Foshan the 14th Five-Year Plan Key Discipline Foundation, the Guangdong Provincial TCM Bureau Key Discipline Foundation, and the Foshan Brain Heart Co-Therapy Foundation. The Foshan 14th Five-Year Priority Speciality Construction Project, The Foshan Nanhai District 14th Five-Year Priority Speciality Construction Project, Foshan Traditional Chinese Medicine Immune Health Technology Innovation Base.

## Conflict of interest

The authors declare that the research was conducted in the absence of any commercial or financial relationships that could be construed as a potential conflict of interest.

The handling editor declared a shared affiliation with the author EJ at the time of review.

## Publisher’s note

All claims expressed in this article are solely those of the authors and do not necessarily represent those of their affiliated organizations, or those of the publisher, the editors and the reviewers. Any product that may be evaluated in this article, or claim that may be made by its manufacturer, is not guaranteed or endorsed by the publisher.
